# Stigma towards people with tuberculosis: a cross-cultural adaptation and validation of a scale in Indonesia

**DOI:** 10.1186/s40359-023-01161-y

**Published:** 2023-04-13

**Authors:** Ahmad Fuady, Bustanul Arifin, Ferdiana Yunita, Saidah Rauf, Agus Fitriangga, Agus Sugiharto, Finny Fitry Yani, Helmi Suryani Nasution, IWayan Gede Artawan Eka Putra, Muchtaruddin Mansyur, Tom Wingfield

**Affiliations:** 1 Department of Community Medicine, Faculty of Medicine, Universitas Indonesia, Pegangsaan Timur No 16, Jakarta, 10310 Indonesia; 2 Primary Health Care Research and Innovation Center, Indonesian Medical Education and Research Institute, Faculty of Medicine, Universitas Indonesia, Jakarta, 10430 Indonesia; 3 Department of Public Health, Erasmus MC University Medical Center Rotterdam, 3015CN Rotterdam, The Netherlands; 4 Faculty of Pharmacy, Universitas Hasanuddin, Makassar, Sulawesi Selatan 90245 Indonesia; 5 Unit of Global Health, Department of Health Sciences, University of Groningen, University Medical Centre Groningen (UMCG), Ant. Deusinglaan 1, 9713 AV Groningen, The Netherlands; 6 Department of Health Behaviour, Environment, and Social Medicine, and Centre of Health Behaviour and Promotion, Faculty of Medicine, Public Health and Nursing, Universitas Gadjah Mada, Yogyakarta, Indonesia; 7 Department of Community Medicine, Faculty of Medicine, Universitas Gunadarma, Depok, 16451 Indonesia; 8 Politeknik Kesehatan Kemenkes Maluku, Maluku, 97711 Indonesia; 9 Department of Community Medicine, Faculty of Medicine, Universitas Tanjungpura, Pontianak, 78124 Indonesia; 10 Department of Child Health, Faculty of Medicine, Universitas Andalas, Padang, 25129 Indonesia; 11 Department of Paediatric, Dr. M. Djamil General Hospital, Padang, 25128 West Sumatera Indonesia; 12 Faculty of Medicine and Health Sciences, Universitas Jambi, Jambi, 36122 Indonesia; 13 Department of Public Health and Prevention Medicine, Faculty of Medicine, Universitas Udayana, Bali, 80232 Indonesia; 14 South East Asian Ministers of Education Organization Regional Center for Food and Nutrition, Jakarta, 13120 Indonesia; 15 Department of Clinical Sciences and International Public Health, Liverpool School of Tropical Medicine, Liverpool, L3 5QA UK; 16 Department of Global Public Health, WHO Collaborating Centre on Tuberculosis and Social Medicine, Karolinska Institute, 171 76 Stockholm, Sweden; 17 Tropical and Infectious Disease Unit, Royal Liverpool and Broadgreen University Hospitals NHS Trust, Liverpool, L7 8XP UK

**Keywords:** Tuberculosis, Stigma, Tool, Scale, Indonesia

## Abstract

**Introduction:**

Tuberculosis (TB) remains a highly stigmatised disease that can cause or exacerbate mental health disorders. Despite increased awareness of the importance of reducing TB stigma, validated tools to measure TB stigma remain scarce. This study aimed to culturally adapt and validate the Van Rie TB Stigma Scale in Indonesia, a country with the second largest TB incidence worldwide.

**Methods:**

We validated the scale in three phases: translation, cultural adaptation, and psychometric evaluation. We invited diverse experts to an interdisciplinary panel for the cross-cultural adaptation, then performed a psychometric evaluation of the scale: exploratory and confirmatory factor analyses, reliability analysis, and correlation analysis with Patient Health Questionnaire 9 [PHQ-9].

**Results:**

We culturally adapted the original scale's language and content during the translation and cultural adaptation phases. After psychometric evaluation with 401 participants in seven provinces of Indonesia, we removed two items. The new scale had two forms: (A) patient and (B) community perspective forms. Both forms had good internal consistency, with respective Cronbach's alpha values of 0.738 and 0.807. We identified three loading factors in Form A (disclosure, isolation, and guilty) and two loading factors in Form B (isolation and distancing). The scale showed correlation with PHQ-9 (Form A, rs = 0.347, *p* < 0.001; Form B, rs = 0).

**Conclusions:**

The culturally adapted Indonesian version of Van Rie's TB Stigma Scale is comprehensive, reliable, internally consistent, and valid. The scale is now ready for applied scale-up in research and practice to measure TB-stigma and evaluate the impact of TB-stigma reduction interventions in Indonesia.

**Supplementary Information:**

The online version contains supplementary material available at 10.1186/s40359-023-01161-y.

## Introduction

Tuberculosis (TB) remains a highly stigmatised and stigmatising disease [1, 2]. People with TB and its associated symptoms and signs, such as cough and weight loss, are often exposed to negative attitudes from people around them [3]. This may take the form of avoidance of being close to someone with TB and can lead to isolation, rejection or exclusion from the community and their workplace, or even from their close family and healthcare staff who provide care for them [4]. All these behaviours, increase the risk of mental health disorders among people with TB. Although some TB-stigma may be grounded in justifiable public health concerns regarding avoidance of TB transmission, much TB-stigma relates to myths and misconceptions about TB, including TB being incurable, hereditary, curse, and spread by non-respiratory routes such as sharing cutlery [5, 6].

TB-stigma has been recognized by the World Health Organization (WHO) [7] and the United Nations [8] as a key barrier to eliminating TB globally. Stigma experienced or anticipated by people having TB-related symptoms can negatively influence their access to TB diagnostic and treatment services leading to delayed diagnosis and suboptimal engagement with care. In this way, TB-stigma can be associated with challenges to treatment adherence, which can reduce TB treatment success [1, 9–11] and increase the risk of TB transmission and the development of drug-resistant TB. More broadly, there is also emerging evidence that TB-stigma can result in mental health disorders [12] and catastrophic out-of-pocket costs and lost income [13, 14].

Given these problems, measuring TB-stigma is critical to understand its prevalence, roots, and determinants, and to assess the effectiveness of potential TB-stigma mitigation strategies [11]. There have been multiple scales and tools developed to assess TB-stigma [15, 16]. However, such scales and tools need to be adapted, validated, piloted, and refined before scale-up in a specific community or population to ensure their accuracy, reliability, and robustness. One of the most widely used tools is Van Rie’s TB Stigma scale [17, 18]. This scale has showed good internal consistency and been validated in various languages and settings including Thailand [17], Portugal [19], Mexico [20], Turkey [21], and Vietnam [22].

Despite measuring and addressing TB-stigma being a vital component to ending TB and relieve mental problems among people with TB, many high TB burden countries still lack locally adapted and validated TB-stigma scales. We aimed to translate, adapt, and validate the Van Rie’s TB Stigma Scale in Indonesia, a middle-income country with the second highest TB incidence worldwide.

## Methods

### Instrument

The original Van Rie’s TB Stigma Scale consists of two main parts: Part A 'Community perspectives toward TB’ with 11 items and Part B 'Patient perspectives toward TB’ with 12 items. In each item, participants are provided with four answer options: strongly disagree (0), disagree (1), agree (2), and strongly agree (3) [17].

**Fig. 1:**
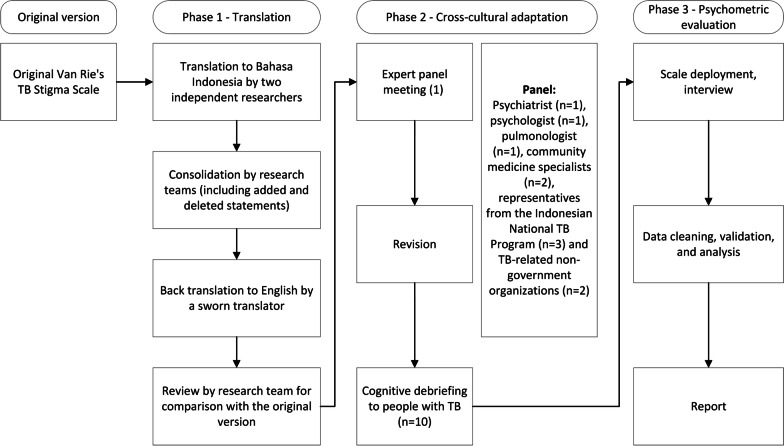
Flow of cross-cultural adaptation and validation of the scale

We translated, adapted, and validated the scale in three consecutive phases from January to July 2022 (Fig. 1). In December 2021, before study initiation, we approached Van Rie and collaborators to seek advice and gained approval to adapt the scale to the Indonesian setting.

### Phase 1: forward translation, consolidation, and backward translation

We followed existing guidance in forward and backward translation [23–25]. We appointed two independent Indonesian doctoral/post-doctoral researchers with experience in TB and community research who have published peer-reviewed scientific articles about TB in high-quality journals, and are fluent in English and Bahasa (the Indonesian lingua franca), to translate the original scale from English into Bahasa. After the translation, we discussed the two translated versions internally, compared the translated versions with the original one, and agreed on one ‘consolidated version’ of the tools. The consolidated version of the tools was backward translated from Bahasa to English by a separate sworn translator who had never seen the original version. We compared the original with the back-translated versions, collectively judged their alignment to be good, and concluded that, despite few literal differences, the translation had the same meaning with the original version.

### Phase 2: cross-cultural adaptation

We purposively invited diverse in-country, interdisciplinary experts: a psychiatrist, a psychologist, a pulmonologist, a community medicine specialist, Indonesian National Tuberculosis Program (NTP) manager, and staffs from TB-related non-governmental organizations to join an advisory panel. The panel provided suggestions to improve the scale, including the clarity of the terms/words used and contextualization to the Indonesian setting.

We revised the translated scale based on the panel meeting and agreed on the pre-final version of the scale in Bahasa. Phrases and terms that had not been completely agreed by the panel were highlighted and further evaluated during cognitive debriefing with 10 adults with TB. We asked the participants whether they understood the items, found ambiguous words or phrases, or felt uncomfortable with any words or items (i.e., items that offended, insulted, or were perceived to be culturally inappropriate). The project team discussed all the suggestions provided by participants, and subsequently revised and finalized the tools for Phase 3.

### Phase 3: Psychometric evaluation

#### Study sites

The study sites were seven provinces of Indonesia: Jakarta, West Sumatra, Jambi (which represented the Western part of Indonesia), South Sulawesi, West Kalimantan, and Bali (Central part), and Maluku (Eastern part). These sites were purposively selected due to TB burden, urban–rural mix, and pre-existing research infrastructure and networks.

#### Participant selection and sample size

In each province, we consulted with NTP officers in province and district levels to purposively select two districts representing an urban and rural area, respectively. We then selected adults with pulmonary drug-susceptible TB (DS-TB), aged ≥ 18 years old, from primary health centres, public hospitals, and private hospitals. We selected participants consecutively from the National TB Program Registers at each health facility from the newest diagnosed, based on their TB treatment status as follows:

A. People receiving TB treatment in the intensive treatment phase (the first two months of a standard six-month DS-TB first treatment regimen) and who had never previously received TB treatment,
B. People who were diagnosed with TB but never started the TB treatment (henceforth termed “lost to follow up to treatment”), or
C. People receiving a six-to-nine-month DS-TB re-treatment regimen, at any phase of treatment.

We selected these respondent groups on the assumption that TB-stigma is experienced during care seeking and early treatment when signs and symptoms of the disease are most obvious and that people who had adverse TB treatment outcomes and had had previous TB episodes were a group at high risk of experiencing TB-stigma. We excluded people with extrapulmonary or (multi) drug-resistant TB (MDR-TB).

There are various recommendations to determine sample size for tool validation [26, 27]. We determined the sample size based on the need to conduct an Alpha Cronbach test and factor analyses. With an assumption that the tools had good internal consistency (Alpha Cronbach of 0.8), confidence level of 95%, and margin of error of 5%, we required at least 246 participants for this validation study [28]. For factor analysis, we assumed that the level of communality was good (0.92). With a wide level of communality and four loading factors, we required at least 240 participants for conducting factor analysis [29].

#### Data collection and statistical analyses

We recruited interviewers with a background in health sciences (medical students, medical doctors, nurses, midwifes or public health graduates) in each province. We then provided a one-day online training to explain the study background, the instruments used, participant selection, and how to ask participants using the instrument.

All interviews were conducted using paper-based questionnaires. After the interview, interviewers entered the data to the RedCap platform (https://redcap.fk.ui.ac.id) for data checking, cleaning, and validation. Data were collected from March 1 to July 31, 2022. Once the data were cleaned and validated, we started data analysis using IBM SPSS 27.0 and RStudio.

#### Floor or ceiling effects

We first performed a descriptive analysis to check the presence of floor or ceiling effects—a condition in which more than 15% of participants choose an item with either the maximum or the minimum score on the scale [30]. Items identified as having a floor or ceiling effect were discussed amongst the project team and considered for exclusion from the scale.

#### Internal consistency

In this study, we applied both exploratory and confirmatory factor analyses to check the scale’s internal consistency. We performed an exploratory factor analysis (EFA) and set a threshold of 0.7 for Kaiser–Meyer–Olkin’s (KMO) and 0.05 for Bartlett's test value to check the fitness of the model [31], and further clarified with Horn's parallel analysis test [32]. Factor analysis was performed by assessing the Eigenvalues ​​using principal axis analysis with varimax rotation to determine the number of factors or domains. We included factors which had eigenvalues > 1, logical and theoretical links between the items, and contained three or more items loading ≥ 0.4. The EFA was performed using IBM SPSS version 25.0. We measured the Cronbach's alpha values for total items and the values per item. A coefficient of > 0.8 was considered to have good to very good internal consistency [33].

A confirmatory factor analysis (CFA) was applied using lavaan package in R. The model was evaluated by calculating Root Mean Square Error of Approximation (RSMEA), Standardized Root Mean Square Residual (SRMR), Comparative Fit Index (CFI) and Tucker-Lewis Index (TLI). RSMEA of less than 0.05 was considered “close” fit and the value of 0.05–0.08 as reasonable model-data fit [34]. The CFI and TFI of more than 0.90 and SRMR of less than 0.08 were also set as thresholds of model fitness [35].

#### Construct validity

In this validation study, we also asked participants the Patient Health Questionnaire (PHQ-9) (Additional file 1: S1). We applied Spearman’s correlation coefficients (*r*_*s*_) to assess the correlations between the loading factors and total stigma scale scores with the total score of PHQ-9. A significant positive correlation between stigma scores and depressive symptoms (*r*_*s*_ = 0.3 to 0.5 for significantly moderate correlation) would indicate that the scale has construct validity and potential discriminatory power to identify depressive symptoms.

### Ethical considerations

This study received a research ethical approval from the Ethics Committee of the Faculty of Medicine, University of Indonesia (No. KET-60/UN2.F1/ETIK/PPM.00.02/2022, on January 17, 2022). Participants received a complete explanation before signing consent to join the interview, and they were allowed to withdraw their participation from this study without any consequences. This study was also supported by the Ministry of Health of the Republic of Indonesia and received research permit from all local health offices in the seven selected Provinces.

### Reporting

Our study design and reporting conforms to the ISPOR principles of translation and cross-cultural adaptation framework [24] (Additional file 1: S2).

## Results

### Participants characteristics

**Fig. 2:**
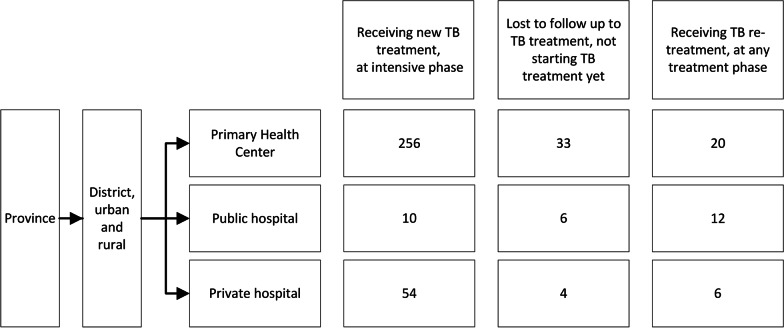
Participant allocation in each facility

We consecutively selected 410 participants. Nine participants refused to join the study, resulting in 401 (98%) participants interviewed, all of whom (100%) completed the interview. Sociodemographic and clinical characteristics of participants are shown in Fig. 2 and Table 1.

**Table 1:** Participants characteristics, n = 401

Characteristics		n	%
*Demographic*			
Sex			
	Male	241	(60.1)
	Female	160	(39.9)
Age in years			
	18–30	108	(26.9)
	31–40	64	(16.0)
	41–50	84	(20.9)
	51–60	80	(20.0)
	> 60	65	(16.2)
Marital status			
	Not married	92	(22.9)
	Married	281	(70.1)
	Widowed, divorced	10	(2.5)
	Widowed, death	18	(4.5)
Educational level			
	No schooling	13	(3.2)
	Elementary school	66	(16.5)
	Junior high school	59	(14.7)
	Senior high school	189	(47.1)
	College/University	74	(18.5)
Provinces			
	Jambi	71	(17.7)
	West Sumatera	33	(8.2)
	Jakarta	31	(7.7)
	West Kalimantan	72	(18.0)
	Bali	31	(7.7)
	South Sulawesi	94	(23.4)
	Maluku	69	(17.2)
Area			
	Urban	271	(67.6)
	Rural	130	(32.4)
*Clinical management*			
Healthcare facility			
	Primary health center (*Puskesmas*)	309	(77.0)
	Public hospital	28	(7.0)
	Private hospital	64	(16.0)
Type of DS-TB treatment			
	First treatment	319	(79.5)
	Retreatment	43	(10.7)
	Not started yet	39	(9.8)
Treatment phase			
	Intensive	252	(62.8)
	Continuation	110	(27.4)
	Lost to follow up to treatment	39	(9.8)

DS-TB, drug-susceptible TB

### Phase 1: translation, consolidation, and back translation

After translation to Bahasa, we did not find significant issues or errors. Following discussion amongst the project team, we decided to reverse the order of the Forms from the Van Rie scale so that ‘Patient perspective toward TB’ came first (Form A) and ‘Community perspective toward TB’ (Form B) follows. This was done because it was perceived that beginning the tool by asking statements using the first-person point of view (i.e., “I feel that….”) was easier to understand and preferable to beginning the tool with the more abstract third person point of view statements (i.e., “Some people feel that…”). Following this consolidated version of the scale, there was no further change, addition, or removal of items and no significant issues were identified after back translation to English.

### Phase 2: cross-cultural adaptation

We provided the expert panel with the consolidated version of the scale in Bahasa and the original version of the scale. The panel suggested some additional refinement to wording to be more appropriate to the Indonesian cultural context. For example, the panel suggest using ‘*kesepian*’ in Bahasa to refer to isolation despite sometimes being translated into English as ‘lonely’. We also changed ‘careless behaviour’ to ‘risky behaviour’ since the change in Bahasa (‘Perilaku ceroboh’ to ‘Perilaku berisiko’) was perceived to be more easily interpreted by participants. The panel did not suggest addition or removal of any scale items.

We tested the pre-final version of the scale with 10 consecutively selected adults with TB in Puskesmas. All participants understood and were able to answer all items. One participant did not know what HIV/AIDS disease is but understood the word after explanation by researcher. One participant was not sure what the word ‘disgusting’ (“menjijikkan” in Bahasa) meant in the context but understood the word after explanation in local dialect. For those two related items, we added explanation notes for interviewers to be used during interviews. Since there was no substantial change from pre-final and final version of the scale, we included the 10 field test responses in the full psychometric evaluation.

### Phase 3: validation with psychometric evaluation

#### Floor and ceiling effect

We identified floor effects on several items (i.e., P2, P3, P5, P7, P10, P11, C18, and C23) and a ceiling effect in only one item (P4). (Table 2) After discussion amongst the project team, we decided to remove P4 from the scale because, in addition to this ceiling effect, it was perceived that the item “I keep my distance from others to avoid spreading TB germs”, could have been temporally influenced by the social distancing and public health measures related to the COVID-19 pandemic rather than specifically TB.

**Table 2:** Floor and ceiling effects in each item

A. Patient perspective			B. Community perspective		
Items	Minimum score (%)	Maximum score (%)	Items	Minimum score (%)	Maximum score (%)
P1	12.2	4.2	C12	15.0	3.7
P2	**17.0**	2.7	C13	4.2	7.7
P3	**19.0**	2.0	C14	5.7	6.0
P4	3.7	**27.2**	C15	10.0	5.0
P5	**27.7**	2.5	C16	5.5	10.7
P6	9.5	7.2	C17	6.5	10.5
P7	**20.0**	3.0	C18	**22.9**	4.7
P8	12.0	11.0	C19	15.0	2.2
P9	6.0	13.5	C20	10.2	5.0
P10	**24.7**	9.2	C21	12.2	4.5
P11	**19.7**	7.5	C22	5.7	7.0
			C23	**20.2**	1.5

P, patient perspective; C, community perspective. Items highlighted in bold are those that exceeded the pre-defined 15% threshold for floor or ceiling effects

#### Internal consistency

The scales had good internal consistency, with a Cronbach's alpha value of 0.738 for Form A (Patient Perspective) and 0.807 for Form B (Community Perspective). There was no single item which significantly reduced the alpha score (Table 3).

**Table 3:** Factor loadings and Cronbach's alpha of the scale

Items	Factor			Mean	Cronbach Alpha if item is deleted
	1	2	3		
Patient perspective toward TB					
P6. I am afraid to tell people outside my family that I have TB	0.780			2.32	0.701
P7. I am afraid to tell others that I have TB because others may think that I also have HIV/AIDS	0.711			2.13	0.717
P9. I choose carefully who I tell about having TB	0.636			2.60	0.709
P12. I am afraid that other people may tell my family that I have TB	0.629			2.09	0.699
P1. I feel hurt by how others react to knowing that I have TB		0.698		2.17	0.720
P2. I have lost friends when I shared with them that I have TB		0.684		2.01	0.711
P3. I feel lonely		0.715		2.00	0.718
P5. I am afraid of going to TB clinics because other people may see me there		0.616		1.90	0.715
P8. I feel guilty because my family has the burden of caring for me			0.516	2.45	0.733
P10. I feel guilty for getting TB because of my smoking, drinking, or other risky behaviours			0.758	2.23	0.745
P11. I am worried about having HIV/AIDS			0.523	2.25	0.736

Community perspective toward TB	Factor		Mean	Cronbach Alpha if item is deleted
	1	2		
C13. Some people may not want to eat or drink with friends who have TB	0.799		2.61	0.858
C14. Some people feel uncomfortable about being near those with TB	0.718		2.63	0.859
C15. If a person has TB, some community members will behave differently towards that person for the rest of his ⁄ her life have HIV/AIDS	0.576		2.04	0.865
C16. Some people do not want those with TB playing with their children	0.747		2.49	0.857
C17. Some people keep their distance from people with TB	0.706		2.36	0.863
C22. Some people may not want to eat or drink with relatives who have TB	0.681		2.69	0.853
C18. Some people think that those with TB are disgusting		0.779	2.56	0.855
C19. Some people do not want to talk to others with TB		0.748	2.33	0.854
C20. Some people are afraid of those with TB		0.757	2.12	0.856
C21. Some people try not to touch others with TB		0.762	2.22	0.859
C23. Some people prefer not to have those with TB living in their community	0.321	0.373	2.01	0.870

In EFA of the 11-item Form A, the KMO value was 0.775, Bartlett's test value was 894.737 (*p* < 0.001) and three distinct factors were identified and characterised: disclosure (P6, P7, P9, and P12), isolation (P1, P2, P3, and P5), and guilt (P8, P10, and P11).

The KMO value of the 11-item Form B was 0.881, Bartlett's test value was 1675.587 (*p* < 0.001), and two factors were identified and characterised: isolation (C13, C14, C15, C,16, C17, and C22) and distancing (C18, C19, C20, and C21). C23 was not conclusive with loading values of 0.321 for factor 1 and 0.373 for factor 2. Because of its inconclusive loading value, the project team decided to exclude item C23 from the scale, leaving the 10-item scale for CFA. Scree plots,reproduced correlations tables, and average variance, composite reliability, and maximum shared variance of TB-Stigma Scale domains are provided in Additional file 1: S3–S6.

#### Confirmatory factor analysis

**Fig. 3:**
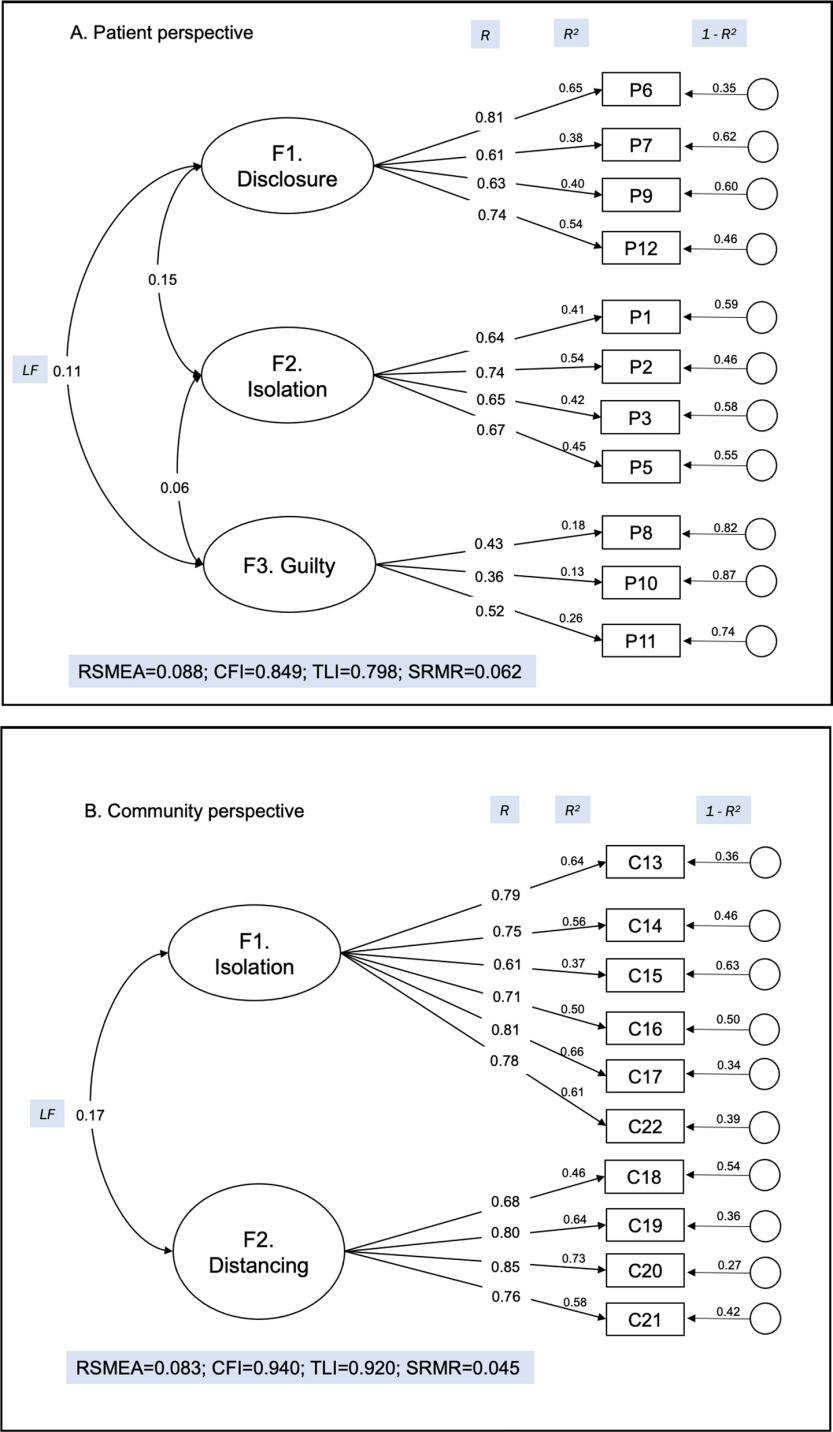
Confirmatory factor analysis of (**A**) Form A: patient perspective and (**B**) Form B: Community Perspective of the TB stigma scale in people with TB in Indonesia. F: loading factors; V: tool’s item; RMSEA: root mean square error of approximation; TLI: Tucker Lewis index; CFI: comparative fit index; LF: covariance between factors; R: variance indicating magnitude of relationship of items to factor; R^2^: percentage of variance of each item explained by factor; 1-R^2^: percentage of variance of each item not explained by factor

In CFA of Form A scale, we found the the scaled (robust) chi-square of *X*^*2*^*(df)* = 169.35(41) for our model (*p* < 0.05). (Fig. 3) The scaled fit indices showed a good fit with the RMSEA value of 0.088, the SRMR of 0.062, CFI of 0.849 and TLI of 0.798. The R^2^ values between loading factors ranged from 0.06 to 0.15. The R^2^ values for each item were uniform for F1 (ranged from 0.61–0.81), F2 (0.64–0.74) and F3 (0.36–0.52).

For Form B, we removed C23 in the CFA. The scaled (robust) chi-square for our model of Form B was *X*^*2*^*(df)* = 127.39 (34) (*p* < 0.05, Fig. 3). The model was a considerably fit in the scaled fit indices, with the RMSEA value of 0.083, the SRMR of 0.045, CFI 0.940. and TLI of 0.920. The R^2^ values between loading factors ranged was 0.17. The R^2^ values for each item were uniform for F1 (ranged from 0.61–0.81) but widely distributed for F2 (0.36–0.78).

#### Construct validity

The reliability of PHQ-9 was good, with Cronbach's alpha of 0.837. We then used the PHQ-9 to assess the construct validity of the Indonesian version of TB-Stigma Scale. Form A had moderate correlation with Form B (rs = 0.416, *p* < 0.001) and PHQ-9 (rs = 0.347, *p* < 0.001, Table 4). Form B had a weak correlation with PHQ-9 (rs = 0.119, *p* < 0.001) but the distancing factor was not significantly correlated with PHQ-9.

**Table 4:** Correlation of Stigma Scale Form A (Patient Perspective) with Form B (Community Perspective) and with PHQ-9 score

Factors’ scores	Community perspective score		PHQ-9 score	
	*r*_*s*_	*P*	*r*_*s*_	*P*
Form A: patient perspective				
Disclosure	0.338	< 0.001	0.254	< 0.001
Isolation	0.351	< 0.001	0.282	< 0.001
Guilt	0.210	< 0.001	0.263	< 0.001
Total stigma score	0.416	< 0.001	0.347	< 0.001
Form B: community perspective				
Isolation			0.240	< 0.001
Distancing			0.080	0.111
Total Stigma Score			0.199	< 0.001

## Discussion

This is the first study adapting and validating Van Rie’s TB Stigma Scale to the Indonesian setting. Despite multiple studies assessing TB-stigma in Indonesia [36–39], none have previously described adaptation and validation of a TB-stigma scale prior to implementation. This new Indonesian version of the scale, consisting of an 11-item Form A (Patient Perspective) and a 10-item Form B (Community Perspective), was found to be reliable, internally consistent, and valid, and fills the knowledge gap.

During validation, two items were removed: “I keep my distance from others to avoid spreading TB germs” (Item P4, Form A) and “Some people prefer not to have those with TB living in their community” (Item C23, Form B). Item P4 showed a ceiling effect, which may have been influenced by increased knowledge and awareness of social distancing and community-level public health measures during the COVID-19 pandemic when this study was conducted [40, 41]. It was perceived that this item could not specifically assess TB stigma. Future studies measuring TB-stigma, therefore, should consider incorporation of items relating to COVID-19 on responses given alterations in societal norms and behaviour since the pandemic began, especially in the South-East Asian Region. Item C23, despite being found to be clear, understandable, and reliable, was not aligned with a specific factor following EFA. The reason behind is unclear. While there is evidence that people with TB may be avoided by others and excluded from certain social activities [42], the evidence is less clear on exclusion, banishment, or being ostracized from communities in the current context of Indonesia rather than the exclusion of people with HIV/AIDS [43] or those from the LGBTQplus community [44].

Despite the low prevalence of HIV in the seven selected provinces and general population of Indonesia [45], the study found that items relating to HIV/AIDS (P7, P11, and C15) were still relevant to participants and perceived to be important to maintain in the scale, as findings from Thailand [17] and Brazil [19]. This is different to a study from Vietnam, which consider the items as of less relevant because of the low local prevalence of TB/HIV co-infection [22]. Importantly, and similar to a study in Mexico [20], our EFA results showed that HIV-related items did not form distinct loading factors. Instead, item P7 was more closely related to the “Disclosure” loading factor and item P11 was more related to the “Guilty”. These findings may indicate that HIV/AIDS is still viewed prejudicially and associated with significant stigma in Indonesian communities.

The new scale we have developed was able to capture several types of TB-stigma. In Form A, the ‘Disclosure’ loading factor represented anticipated stigma, which is an expectation and fear of discrimination and behaviour of others towards people with TB who disclose their TB status. The ‘Isolation’ loading factor is more likely to represent enacted or experienced stigma, which is indicated by feeling hurt, feeling lonely, and losing friends. The ‘Guilty’ loading factor implies self-stigma, indicated by people with TB accepting a negative stereotype about themselves and feeling guilt and shame related to their diagnosis. Our finding of groups of loading factors is helpful because it can enable identification of potential target populations for interventions to reduce TB-stigma and support consideration the mechanisms by which the interventions may be expected to achieve impact [46]. For example, interventions focusing on wide public health communications through mass media may work by reducing enacted stigma, interventions targeted towards people or groups at high risk of TB may work by mitigating anticipated stigma and may eventually reduce delay to TB diagnosis, and interventions among people with TB will focus on relieving self-stigma and associated deterioration in mental health.

Both forms of the Indonesian version of TB Stigma Scale showed good internal consistency. This finding is consistent with findings of validation studies of the Van Rie scale in other settings [17, 19–22]. The overall model fitness, indicated by Chi-Square test, showed a *P*-value of < 0.05, which was encouraging but may have related to large sample size [47]. However, the scaled fitness indices, indicated by RSMEA, CFI, and TLI, showed good fitness supporting the interpretation that the scale was reliable and consistent [31, 34, 35].

Previous published review showed that most studies measuring TB-stigma used disparate, unvalidated tools, which limited interpretation of their results and hindered cross-country comparisons [46]. The findings of our study in Indonesia will contribute to a growing evidence base on locally and culturally appropriate and validated TB stigma measurements tools. Expanded use of such tools will not only be of benefit to the NTP and the people they serve but will also enhance the systematic evaluation of the impact of TB-stigma reduction interventions, which up to now have lacked validated tools [46].

Although we selected participants from seven provinces, given the wide geographical and cultural contexts of Indonesia, it remains vital to assess whether the scale is generalizable nationally or requires further adjustment and refinement to specific populations, cultures, and regions of Indonesia. People living in different geographical areas, speaking different dialects, and having different sets of values, beliefs, experiences, and communication styles in community may influence the interpretation to question items [48]. This study excluded people with DR-TB and focused on people diagnosed with DS-TB because they constitute the majority of people with TB in Indonesia and are at risk of acquired drug-resistance if their adherence is poor. This focus on people with DS-TB may limit the generalizability of our findings and the use of the validated tool amongst people with DR- and MDR-TB in Indonesia.

## Conclusions

We designed, implemented, and evaluated a culturally adapted version of Van Rie’s TB Stigma Scale, which was found to be comprehensive, reliable, internally consistent, and valid in the Indonesian setting. The final scale, which is ready to implement both within research programmes and programmatically, consisted of an 11-item Form A (Patient Perspective) and a 10-item Form B (Community Perspective). The scale could support identification of TB-affected people and communities at greatest risk of stigma and enable evaluation of the impact of TB-stigma reduction interventions in these vulnerable groups.

## Supplementary Information

**Additional file 1.** Supplementary File: S1–S6.

## Data Availability

Data generated or analysed during this study are available and accessible by requesting to corresponding author.
