# Supplementary material for: Stigma towards people with tuberculosis: a cross-cultural adaptation and validation of a scale in Indonesia
Source: BMC Psychol. 2023 Apr 13;11:112. doi: 10.1186/s40359-023-01161-y (PMC10100612; doi:10.1186/s40359-023-01161-y)
Supplement: Supplementary file 1 — Additional file 1. Supplementary File: S1–S6. [file 40359_2023_1161_MOESM1_ESM.docx]

**Supplementary File**

**Supplement 1.** Original English version of PHQ-9

| **PATIENT HEALTH QUESTIONNAIRE-9**  **(PHQ-9)** | | | | |
| --- | --- | --- | --- | --- |
| **Over the last 2 weeks, how often have you been bothered by any of the following problems?** *(Use “*✔*” to indicate your answer)* | **Not at all** | **Several days** | **More than half the days** | **Nearly every day** |
| **1.** Little interest or pleasure in doing things | 0 | 1 | 2 | 3 |
| **2.** Feeling down, depressed, or hopeless | 0 | 1 | 2 | 3 |
| **3.** Trouble falling or staying asleep, or sleeping too much | 0 | 1 | 2 | 3 |
| **4.** Feeling tired or having little energy | 0 | 1 | 2 | 3 |
| **5.** Poor appetite or overeating | 0 | 1 | 2 | 3 |
| **6.** Feeling bad about yourself — or that you are a failure or have let yourself or your family down | 0 | 1 | 2 | 3 |
| **7.** Trouble concentrating on things, such as reading the newspaper or watching television | 0 | 1 | 2 | 3 |
| **8.** Moving or speaking so slowly that other people could have noticed? Or the opposite — being so fidgety or restless that you have been moving around a lot more than usual | 0 | 1 | 2 | 3 |
| **9.** Thoughts that you would be better off dead or of hurting yourself in some way | 0 | 1 | 2 | 3 |

**FOR OFFICE CODING**  *0*  **+** ______ **+** ______ **+** ______

**=Total Score:** ______

**Supplement 2.** Checklist of the ISPOR principles of translation and cross-cultural adaptation framework in this study

| **No** | **Steps** | **Done** | **Notes** |
| --- | --- | --- | --- |
| 1 | **Preparation** |  |  |
|  | Obtain permission to use instrument | Yes | Permitted by Van Rie and team |
|  | Invite instrument developer to be involved | Yes | A guidance for instrument was sent by Van Rie and team |
|  | Develop explanation of concepts in instrument | Yes |  |
|  | Recruit key in-country persons to the project | Yes | Invited to panel expert |
| 2 | **Forward Translation** |  |  |
|  | Development of at least two independent forward translations | Yes | With two independent translators |
|  | Provision of explanation of concepts in the instrument to the key in-country persons and forward translators | Yes | Presented at expert panel meeting |
| 3 | **Reconciliation of the forward translations** into a single forward translation | Yes | In this study, we used term “Consolidation” |
| 4 | **Back translation** of the reconciled translation into the source language | Yes |  |
| 5 | **Back translation review**: Review of the back translations against the source language | Yes | Done by research team |
| 6 | **Harmonization** of all new translations with each other and the source version | Yes | Done by research team |
| 7 | **Cognitive debriefing** of the new translation, usually with patients drawn from the target population | Yes | With 10 people with TB |
| 8 | **Review of cognitive debriefing** results and finalization | Yes | Done with research team |
| 9 | **Proof reading**: The finalized translation is proofread | Yes | Research team checked the final translation and corrected any remaining spelling, diacritical, grammatical, or other errors |
| 10 | **Final report**: is written on the development of the translation | Yes |  |


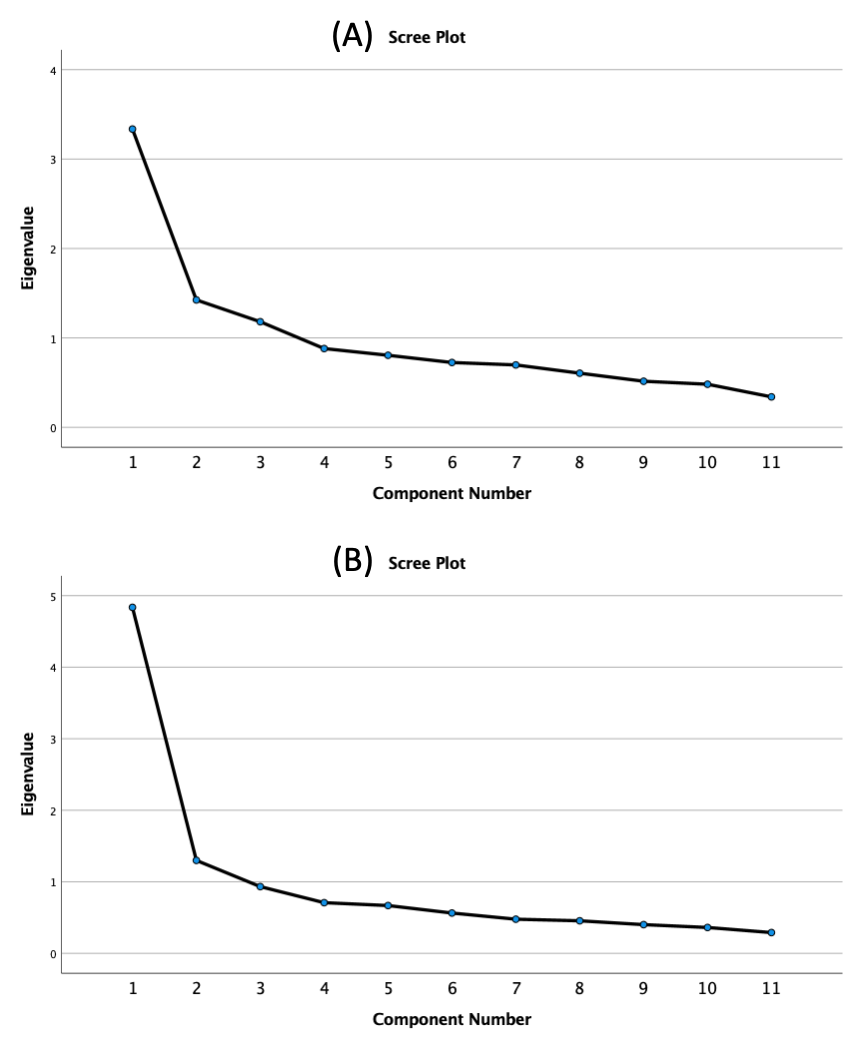


**Supplement 3.** Scree Plot for (A) Form A: Patient Perspective and (B) Form B: Community Perspective

**
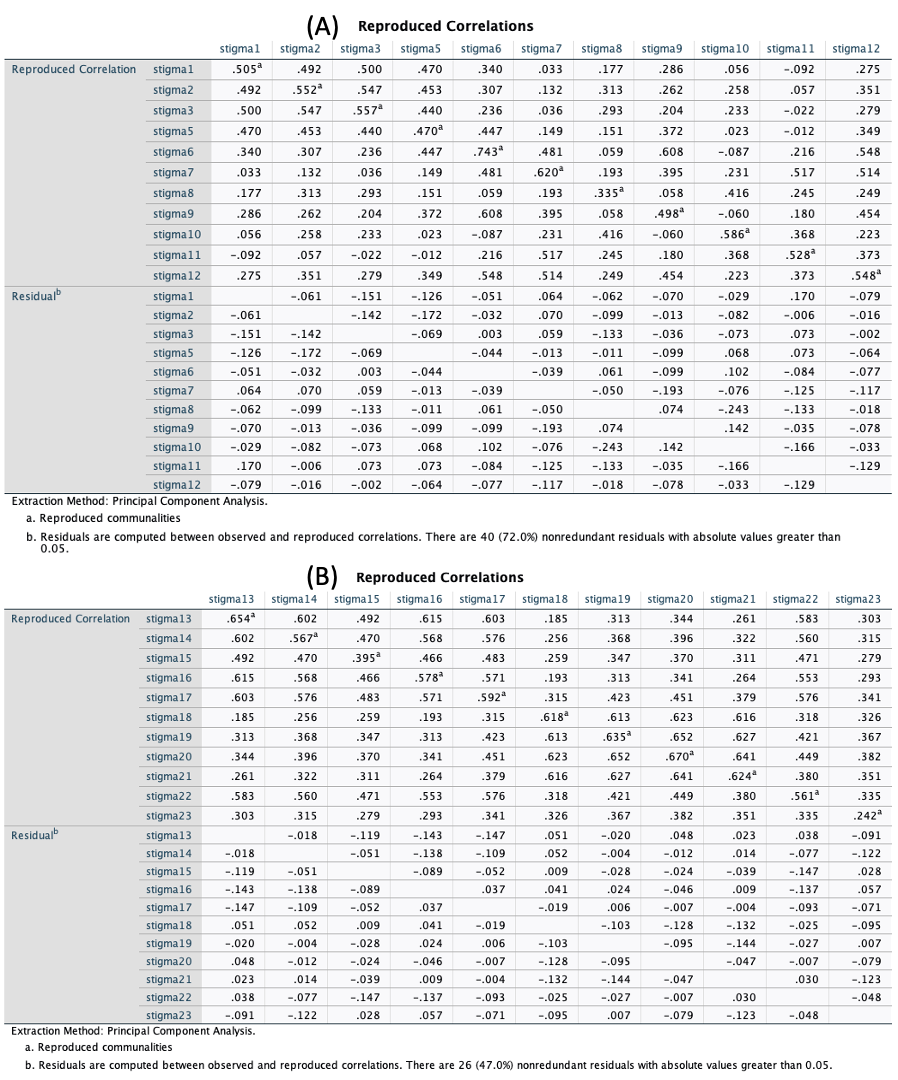
**

**Supplement 4.** Reproduced correlations for (A) Form A: Patient Perspective and (B) Form B: Community Perspective

**Supplement 5.** Horn’s parallel analysis

| **Component** | **Principal component analysis** | | | **Parallel analysis** | |  |
| --- | --- | --- | --- | --- | --- | --- |
|  | **Initial Eigenvalues** | | | **Random Data Eigenvalues** | | |
|  | **Total** | **% of Variance** | **Cumulative %** | **Root** | **Means** | **Percentile** |
| **Form A: Patient Perspective** | | | |  |  |  |
| 1 | 3.336 | 30.328 | 30.328 | 1 | 1.273775 | 1.35177 |
| 2 | 1.425 | 12.957 | 43.285 | 2 | 1.19732 | 1.24339 |
| 3 | 1.181 | 10.740 | 54.025 | 3 | 1.133904 | 1.17004 |
| 4 | .881 | 8.013 | 62.039 | 4 | 1.084146 | 1.126101 |
| 5 | .806 | 7.330 | 69.369 | 5 | 1.038353 | 1.077054 |
| 6 | .725 | 6.595 | 75.964 | 6 | 0.993232 | 1.022189 |
| 7 | .698 | 6.348 | 82.313 | 7 | 0.947996 | 0.982078 |
| 8 | .606 | 5.509 | 87.822 | 8 | 0.905221 | 0.938285 |
| 9 | .516 | 4.691 | 92.512 | 9 | 0.859812 | 0.905595 |
| 10 | .482 | 4.386 | 96.898 | 10 | 0.809883 | 0.845185 |
| 11 | .341 | 3.102 | 100.000 | 11 | 0.756358 | 0.802062 |
| **Form B: Community Perspective** | | | |  |  |  |
| 1 | 4.468 | 44.679 | 44.679 | 1 | 1.256404 | 1.32113 |
| 2 | 1.173 | 11.728 | 56.406 | 2 | 1.179502 | 1.228016 |
| 3 | .900 | 9.002 | 65.408 | 3 | 1.116668 | 1.151042 |
| 4 | .708 | 7.083 | 72.491 | 4 | 1.065396 | 1.100054 |
| 5 | .664 | 6.644 | 79.135 | 5 | 1.016058 | 1.046303 |
| 6 | .528 | 5.283 | 84.419 | 6 | 0.97068 | 1.001144 |
| 7 | .469 | 4.695 | 89.114 | 7 | 0.921546 | 0.948367 |
| 8 | .419 | 4.193 | 93.307 | 8 | 0.877029 | 0.90852 |
| 9 | .366 | 3.655 | 96.962 | 9 | 0.82924 | 0.873758 |
| 10 | .304 | 3.038 | 100.000 | 10 | 0.767477 | 0.819032 |
| Extraction Method: Principal Component Analysis. | | | |  |  |  |

In Form A: Patient Perspective, the random eigen values (see ‘percentile’ column) are lower than initial Eigenvalues (see ‘total’ column) for component 1, 2, 3, then we can conclude that Form A has three loading factors.

In Form B: Community Perspective, the random eigen values (see ‘percentile’ column) are lower than initial Eigenvalues (see ‘total’ column) for component 1, we can conlude that Form B has certainly one loading factor. The second component is at borderline, but we decided to include it as the second loading factor.

**Supplement 6.** Average variance, composite reliability, and maximum shared variance of TB-Stigma Scale domains.

| **Domain** | | **AVE** | | **CR** | | **MSV** |
| --- | --- | --- | --- | --- | --- | --- |
| Form A: Patient Perspective | |  | |  | |  |
|  | Disclosure | | 0.479 | 0.78 | 0.608 | |
|  | Isolation | | 0.461 | 0.77 | 0.511 | |
|  | Guilt | | 0.279 | 0.63 | 0.575 | |
| Form B: Community Perspective | |  | |  | |  |
|  | Isolation | | 0.511 | 0.90 | 0.638 | |
|  | Distancing | | 0.580 | 0.84 | 0.607 | |

**AVE,** average variance; **CR,** composite reliability; **MSV,** maximum shared variance
